# Conicity index increases the risk of female infertility: An analysis of NHANES 2013 to 2018

**DOI:** 10.1097/MD.0000000000044457

**Published:** 2025-09-12

**Authors:** Jia-Yi Zhao, Meng-Hui Hong, Xiao-Ming Yang, Tao Zhou, Shao-Cong Liang

**Affiliations:** a Department of Clinical Laboratory, The Affiliated Foshan Women and Children Hospital, Guangdong Medical University, Foshan, Guangdong, China; b Department of Laboratory Medicine, Zhujiang Hospital, Southern Medical University, Guangzhou, China.

**Keywords:** body mass index, conicity index, infertility, NHANES, relative fat mass, waist-to-height ratio

## Abstract

This study investigates the association between anthropometric indices and infertility in women of childbearing age. : The original data of women aged 20 to 45 years were obtained from National Health and Nutrition Examination Survey (NHANES) 2013 to 2018 for the present cross-sectional study. Data of reproductive status was defined according to answer to Reproductive Health Questionnaire. Each participant’s anthropometric indices were calculated according to the formulas. Weighted logistic regression analysis, sensitivity analysis, subgroup analysis, smooth curve fitting and receiver-operating characteristic curve analysis were employed to investigate the association between anthropometric indices and infertility. A total of 2948 females aged 20 to 45 years were included and analyzed, with 337 (11.43%) self-reported infertility. Weighted logistic regression analysis linked conicity index (C-index) to infertility after adjusting for potential confounders, with an OR of 35.95 (95% CI: 6.50–198.95). Compared with the lowest tertile (T1), the third tertile (T3) of C-index was positively correlation with infertility, with an OR of 2.21 (95% CI: 1.47–3.33). Subgroup analysis showed that C-index tended to be associated with infertility in all participants aged 20 to 45 years. Smoothed curve fitting showed a positive linear relationship between C-index and infertility. Compared with other obesity indicators, C-index (AUC = 0.608) also shows good predictive performance for infertility. These differences were statistically significant (all *P* < .05). Our study showed that C-index had a stronger connection with the risk of infertility than other markers of obesity including body mass index (BMI), relative fat mass (RFM) and waist-to-height ratio (WHTR), and managing obesity as determined by C-index may help to reduce the risk of infertility.

## 1. Introduction

Infertility is generally defined as the inability to conceive in a couple with regular sexual activity without using any contraception for more than 12 months. Infertility affects millions of reproductive aged-women, and according to study, the rate of infertility ranges from 8% to 12% worldwide.^[1,2]^ Approximately 1 in 7 couples in developed countries and 1 in 4 couples in developing countries suffer from infertility.^[3,4]^ Infertility, as a serious global public health concern, does harm to the physical and mental health for couple of reproductive age. However, the determinants of infertility have not been fully elucidated in existing studies.

More and more evidence emphasized the adverse impact of lifestyle related risks, including age, exercise, smoking, alcohol consumption, obesity and psychological stress on infertility.^[5,6]^ Obesity emerged as an increasingly common among women in modern society.^[7–9]^ There were more than 20% of American women of reproductive age struggled with obesity according to the World Health Organization.^[9]^ Obesity has been proved not only to increase the risk of common disease, such as hypertension and diabetes, but also link to irregular menstruation, impaired ovulation, and endometrial pathology, which result in female infertility.^[4,10–14]^

Body mass index (BMI), the most commonly used benchmark for measure of obesity, was proved to link to infertility. Zhu et al showed that too low or too high of BMI was a potential hazard for infertility in women.^[15]^ However, BMI is limited in its ability to differentiate between fat mass and muscle mass, and do not accurately reflect fat distribution of individual.^[3,16,17]^ BMI appears to be insensitive to abdominal fat deposition. Thus, several anthropometric indices sensitive to provide body fat distribution have been proposed, including conicity index (C-index), relative fat mass (RFM) and waist-to-height ratio (WHTR).

The C-index, first described by Valdez et al, proposed based on the situation that individuals who accumulate more fat around the abdomen are biconical, while those have less fat around in the central region are cylindrical. C-index are calculated by simple variables, including weight, height and waist circumference (WC). C-index is a good tool to assess fat distribution, and its value increases with the accumulation of fat in the abdominal region.^[18]^ Compared with BMI and WC, C-index can not only assess fat mass in obese individuals, but also evaluate abdominal fat mass in thin individuals.^[19]^ Previous research indicated that C-index advantage in discriminate underlying abdominal obesity.^[20,21]^ Moreover, C-index is proved to be associated with cardiovascular disease, changes in blood glucose, blood triglyceride and blood pressure.^[22,23]^

Other anthropometric indices such as WC, RFM, and WHTR have been used as clinical measures of central obesity.^[24]^ RFM is a reliable index for estimating whole-body fat percentage based on the height-to-WC and biological sex. Woolcott et al demonstrated that RFM could better reflect the whole-body fat percentage than BMI.^[25]^ WHTR is the ratio of WC to height, and is the more effective measure for abdominal obesity than WC.^[26–29]^ Research has been reported that the WHTR is a reliable indicator for predicting T2DM and a high WHTR was also related to cardiovascular disease risk.^[30–32]^

Previous studies have underlined a close relationship between obesity and infertility. Better simple indicators that can accurately reflect an individual’s fat distribution are needed to identify women at risk of adverse reproductive outcomes. It is necessary to identify the association between obesity estimated by anthropometric indices and infertility. Therefore, we explore the association between C-index and infertility in the US female by using the data from National Health and Nutrition Examination Survey (NHANES) 2013 to 2018. We hypothesized that C-index increases the risk of female infertility.

## 2. Materials and methods

### 2.1. Data source

The original data for this study were obtained from the NHANES, a program that began in the early 1960s. NHANES is a national research program combining interviews and physical examinations run by the National Center for Health Statistics (NCHS) to assess the health and nutritional status of the civilian, non-institutionalized population in the United States. It was conducted by using a sophisticated, multistage sampling design to select a nationally representative sample of about 5000 participants each year. Every 2 years, NHANES letters comprehensive data consisting of questionnaire, physical and biological examination about 10,000 individuals across the country. The program was approved by the Research Ethics Review Committee of NCHS and all survey participants signed their written informed permission. On the official website (https://www.cdc.gov/nchs/nhanes/), all detailed NHANES research designs and data are free of charge to be obtained.

### 2.2. Study participants

**Figure 1.**
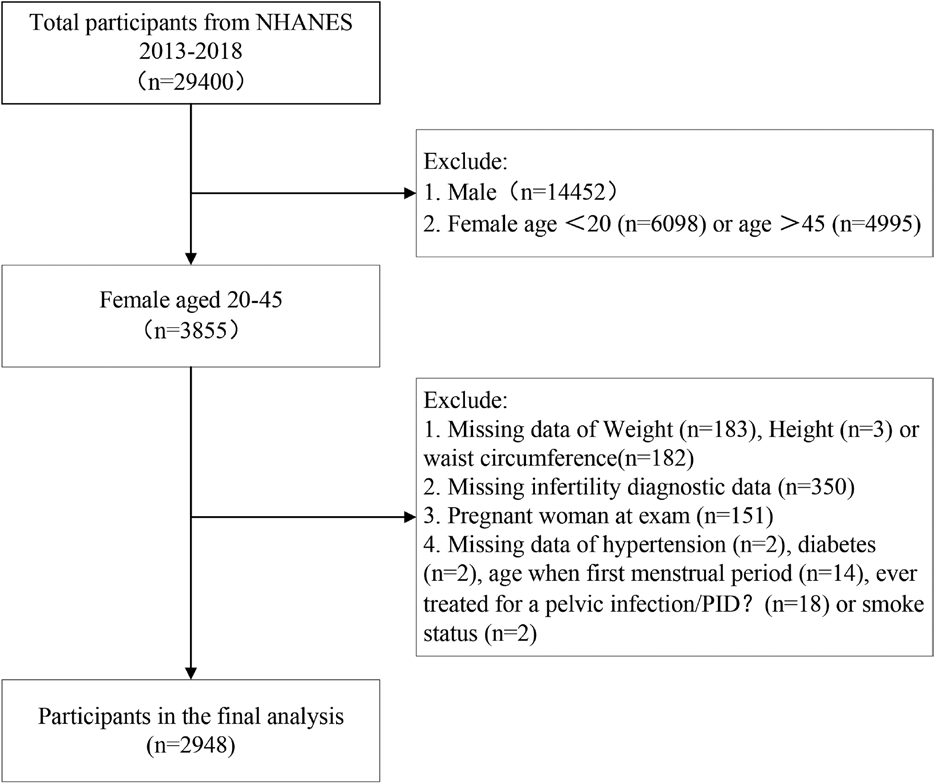
Flowchart of participants selection from NHANES 2013–2018. NHANES = National Health and Nutrition Examination Survey.

Data were drawn from the NHANES collected between 2013 and 2018. A total of 29,400 participants were screened in the NHANES survey over that period. We excluded male participants (n = 14,452), women older than 45 years (n = 4995) and younger than 20 years (n = 6098). Similarly, those with missing data of the weight (n = 183), height (n = 3) or WC (n = 182), without completing the infertility questionnaire (n = 350), pregnant woman at examination (n = 151), missing data of hypertension (n = 2), diabetes (n = 2), age when first menstrual period (n = 14) and was used to diagnose PID (n = 18) or smoke status (n = 2) were also excluded. Ultimately, 2948 eligible female participants were included in final analysis (Fig. 1).

### 2.3. Calculation of the BMI, C-index, RFM, and WHTR

Body weight, height and WC were measured by trained health technicians in a mobile examination center. BMI (Equation 1), C-index (Equation 2), RFM (Equation 3), and WHTR (Equation 4) were calculated with the following formulas^[33,34]^:

$$\begin{array}{l} BMI=\frac{weight\left(kg\right)}{{\left[height\left(m\right)\right]}^{2}} \end{array}$$ (1)

$$\begin{array}{l} C-index=0.109^{-1}WCm){\left[\frac{weight\left(kg\right)}{height\left(m\right)}\right]}^{-1/2} \end{array}$$ (2)

$$\begin{array}{l} RFMwomen=76-\left[20\times \left(\frac{height\left(m\right)}{waist\left(m\right)}\right)\right] \end{array}$$ (3)

$$\begin{array}{l} WHTR=\frac{WC\left(m\right)}{height\left(m\right)} \end{array}$$ (4)

The BMI was each divided into 3 groups: “Underweight or Normal” (<25 kg/m^2^), “Overweight” (≥25, <30 kg/m^2^), and “Obesity” (≥30 kg/m^2^). The eligible participants were divided into 3 groups according to their C-index, RFM, and WHTR tertiles for subsequent analysis, and additionally these anthropometric indices were treated as a continuous variable in the analysis.

### 2.4. Definition of infertility

Infertility was defined according to answer to the specific question in Questionnaire RHQ074: “Have you ever attempted to become pregnant over a period of at least a year without becoming pregnant?” Any woman whose answer was “yes” to the question was considered as infertility.

### 2.5. Assessment of covariates

According to previous literature or clinical experience, and available data in the NHANES, additional covariates including age (20–45 years), race (Mexican-American, Other Hispanic, Non-Hispanic White, Non-Hispanic Black, other race), education level (Less than high school, high school, More than high school), marital status (Married or Living with partner, Widowed/divorced/separated, Never married), income to poverty ratio (PIR), total cholesterol (Chol), uric acid (UA), consuming alcohol status (based on the question “In the past 12 months, how often did you drink any type of alcoholic beverage?), smoking status (based on the questions “Have you smoked at least 100 cigarettes in your entire life?” and “Do you now smoke cigarettes?”), hypertension, diabetes, age of menarche, and pelvic inflammatory disease (PID) (based on the question “Have you ever been treated for an infection in your fallopian tubes, uterus or ovaries, also called a pelvic infection, pelvic inflammatory disease, or PID?”) were selected.^[35–39]^

### 2.6. Statistical analysis

Data description and statistical analysis were based on complex NHANES sampling weights (https://www.cdc.gov/nchs/nhanes/index.html). We described continuous variables using the mean with standard error and categorical parameters were presented as survey-weighted percentage (95% CI). Missing values of PIR (n = 228), Chol (n = 123), UA (n = 124) were estimated by mean value.

We performed survey-weighted linear regression to analyze the characteristics of study population for continuous variables, while survey-weighted Chi-square test were used for categorical variables. The weighted multivariable logistic regression was used to analyze the risk relationship between anthropometric indices and infertility by constructing 3 models: crude model was unadjusted; model I was adjusted for age, race and education level; model II was further adjusted for age, race, education level, marital status, PIR, Chol, UA, hypertension, diabetes, ever treated for a pelvic infection/PID, drinking, smoking, age when first menstrual period occurred.

To explore relationship between anthropometric indices with infertility, 3 main steps were used in our statistical analyses. First, the continuous variable anthropometric indices were transformed into a categorical variable for sensitivity analysis. And then the subgroup analyses and interaction tests were performed to ensure the robustness of the result. Third, smooth curve fittings were employed to determine whether the relationship between anthropometric indices and infertility is linear. Finally, receiver-operating characteristic (ROC) curve and the area under the comparison curve (AUC) value were used to predict infertility. PackageR (http://www.r-project.org) and EmpowerStats (https://www.empowerstats.net/en/) were used for all analyses. Statistical significance was assessed at a 2-sided value of *P* < .05.

## 3. Results

### 3.1. Baseline characteristics of study participants

Figure 1 shows the inclusion and exclusion criteria of study participants, and a total of 2948 women aged 20 to 45 years were enrolled in this study, of whom 11.43% (337/2948) were infertile. The baseline characteristics of the selected female participants are presented in Table 1. Compared to the non-infertile women, infertile women were older (35.62 years vs 32.05 years), were married/living with partner (77.09% vs 56.58%), had higher levels of Chol (4.82 mmol/L vs 4.69 mmol/L) and UA (283.99 μmol/L vs 269.40 μmol/L), had higher proportion of hypertension (20.81% vs 12.82%), diabetes (7.17% vs 2.98%) and PID history (9.68% vs 4.24%), had a higher weight (85.93 kg vs 76.66 kg), height (163.69 cm vs 162.52 cm) and WC (103.08 cm vs 94.63 cm). Besides, significant differences were observed among infertile female in BMI (31.99 kg/m^2^ vs 28.98 kg/m^2^), C-index (1.31 vs 1.27), RFM (43.07 vs 40.51), and WHTR (0.63 vs 0.58). There were no statistical differences in race, education level, PIR, alcohol consumption and smoke status.

**Table 1:** Weighted baseline characteristics of participants* (N = 2948).

	Non-infertility	Infertility	*P*-value
No. of participants	2611	337	
Age, yr	32.05 (31.62, 32.48)	35.62 (34.62, 36.62)	<.0001
Race, %			
Mexican-American	11.78 (9.14, 15.06)	11.20 (7.20, 17.02)	.2364
Other Hispanic	8.00 (6.54, 9.75)	5.75 (3.40, 9.56)	
Non-Hispanic White	56.05 (51.10, 60.88)	62.19 (53.58, 70.08)	
Non-Hispanic Black	13.19 (10.59, 16.32)	12.35 (9.18, 16.42)	
Other Race	10.98 (9.29, 12.93)	8.52 (5.90, 12.15)	
Education level, %			
Less than high school	11.19 (9.48, 13.17)	9.81 (6.97, 13.63)	.6988
High school graduate/GED or equivalent	19.09 (16.67, 21.76)	20.53 (15.08, 27.30)	
More than high school	69.72 (65.88, 73.31)	69.67 (63.13, 75.50)	
Marital status, %			
Married/living with partner	56.58 (53.72, 59.39)	77.09 (71.68, 81.73)	<.0001
Widowed/divorced/separated	10.53 (8.82, 12.53)	10.69 (7.20, 15.59)	
Never married	32.89 (30.22, 35.68)	12.22 (9.17, 16.11)	
PIR, %	2.64 (2.52, 2.77)	2.78 (2.54, 3.01)	.2559
Chol (mmol/L)	4.69 (4.64, 4.75)	4.82 (4.69, 4.94)	.0448
UA (μmol/L)	269.40 (266.02, 272.78)	283.99 (274.80, 293.17)	.0041
Alcohol use in the past years, %			
No	7.35 (6.22, 8.68)	11.89 (8.16, 17.01)	.0541
Yes	80.00 (77.03, 82.67)	78.01 (71.14, 83.63)	
Missing	12.65 (10.55, 15.09)	10.10 (6.17, 16.10)	
Smoke status, %			
Smoker	19.52 (17.46, 21.76)	24.60 (18.80, 31.50)	.0633
Nonsmoker	68.35 (65.51, 71.06)	60.88 (54.34, 67.06)	
Ex-smoker	12.13 (10.37, 14.14)	14.52 (10.28, 20.10)	
Hypertension, %			
Yes	12.82 (11.20, 14.64)	20.81 (15.65, 27.14)	.0026
No	87.18 (85.36, 88.80)	79.19 (72.86, 84.35)	
Diabetes, %			
Yes	2.98 (2.38, 3.73)	7.17 (4.79, 10.61)	.0022
No	95.64 (94.73, 96.40)	90.44 (85.95, 93.61)	
Borderline	1.38 (0.99, 1.93)	2.39 (0.91, 6.12)	
Age when first menstrual period occurred (yr)	12.60 (12.52, 12.68)	12.50 (12.26, 12.75)	.4485
Ever treated for a pelvic infection/PID, %			
Yes	4.24 (3.28, 5.46)	9.68 (6.27, 14.68)	.0002
No	95.76 (94.54, 96.72)	90.32 (85.32, 93.73)	
Weight (kg)	76.66 (75.35, 77.96)	85.93 (82.28, 89.59)	.0001
Height (cm)	162.52 (162.08, 162.96)	163.69 (162.72, 164.66)	.0337
Waist circumference (cm)	94.63 (93.47, 95.79)	103.08 (100.15, 106.01)	<.0001
BMI (kg/m^2^)	28.98 (28.47, 29.48)	31.99 (30.63, 33.35)	.0003
BMI (kg/m^2^), %			
<25	37.57 (34.49, 40.75)	28.42 (22.47, 35.22)	<.0001
≥25, <30	25.20 (23.41, 27.07)	16.88 (12.35, 22.64)	
≥30	37.23 (34.72, 39.81)	54.71 (46.63, 62.55)	
C-index	1.27 (1.26, 1.28)	1.31 (1.30, 1.32)	<.0001
C-index tertile, %			
T1	36.47 (34.27, 38.72)	19.03 (14.37, 24.76)	<.0001
T2	33.47 (31.20, 35.81)	31.34 (26.95, 36.09)	
T3	30.07 (26.99, 33.33)	49.63 (43.34, 55.93)	
RFM	40.51 (40.08, 40.94)	43.07 (42.14, 44.00)	<.0001
RFM tertile, %			
T1	38.10 (35.17, 41.12)	23.23 (17.29, 30.46)	<.0001
T2	32.31 (29.94, 34.78)	29.77 (23.48, 36.93)	
T3	29.59 (26.54, 32.83)	47.00 (38.78, 55.38)	
WHTR	0.58 (0.58, 0.59)	0.63 (0.61, 0.65)	<.0001
WHTR tertile, %			
T1	38.10 (35.17, 41.12)	23.23 (17.29, 30.46)	<.0001
T2	32.31 (29.94, 34.78)	29.77 (23.48, 36.93)	
T3	29.59 (26.54, 32.83)	47.00 (38.78, 55.38)	

BMI = body mass index, C-index = conicity index, Chol = cholesterol, PID = pelvic inflammatory disease, PIR = ratio of family income to poverty, RFM = relative fat mass, UA = uric acid, WHTR = waist-to-height ratio.

* For continuous variables: survey-weighted mean (95% CI), *P*-value was by survey-weighted linear regression (svyglm). For categorical variables: survey-weighted percentage (95% CI), *P*-value was by survey-weighted Chi-square test (svytable).

### 3.2. The relationship between BMI, C-index, RFM, WHTR and infertility

The weighted multivariable logistic regression analysis was used to explore the relationship between anthropometric indices and infertility. Our findings indicated that the association between C-index and infertility demonstrated the most significant compared with BMI, RFM and WHTR after adjusting for all confounding factors. As shown in Table 2, participants with a unit higher C-index is related with 35.95 times increase in the risk of infertility (Model II: OR = 35.95, 95% CI: 6.50–198.95), while participants with a unit higher in the BMI RFM, WHTR is related with 3%, 5%,10.09 times increase in the risk of infertility, respectively (Model 3: OR = 1.03, 95% CI: 1.00–1.05; OR = 1.05, 95% CI: 1.01–1.08; OR = 10.09, 95% CI: 1.95–52.10). Sensitivity analysis found that C-index, RFM, WHTR categorized as tertiles, association with the risk of infertility still maintained its statistical significance, while BMI showed no statistical significance. Notably, individuals in the highest C-index tertile had an importantly 221% increased risk compared to participants in the lowest C-index tertile (OR = 2.21, 95% CI: 1.47–3.33).

**Table 2:** Weighted multivariate logistic regression model analysis of BMI, C-index, RFM, WHTR and infertility in all participants.

Outcome: infertility	Crude model		Model I		Model II	
	OR (95%CI)	*P*-value	OR (95%CI)	*P*-value	OR (95%CI)	*P*-value
BMI	1.04 (1.02, 1.06)	.0001	1.03 (1.01, 1.06)	.0020	1.03 (1.00, 1.05)	.0330
BMI (kg/m^2^)						
<25	Reference		Reference		Reference	
≥25, <30	0.89 (0.58, 1.34)	.5690	0.81 (0.52, 1.24)	.3362	0.73 (0.46, 1.14)	.1796
≥30	1.94 (1.33, 2.83)	.0012	1.73 (1.14, 2.63)	.0138	1.42 (0.91, 2.23)	.1360
* P* for trend		.0012		.0110		.1037
C-index	148.61 (37.59, 587.59)	<.0001	74.20 (17.16, 320.73)	<.0001	35.95 (6.50, 198.95)	.0004
C-index tertile						
T1	Reference		Reference		Reference	
T2	1.79 (1.27, 2.53)	.0018	1.58 (1.11, 2.25)	.0153	1.47 (1.01, 2.14)	.0546
T3	3.16 (2.19, 4.57)	<.0001	2.65 (1.81, 3.87)	<.0001	2.21 (1.47, 3.33)	.0010
* P* for trend		<.0001		<.0001		.0007
RFM	1.07 (1.04, 1.10)	<.0001	1.06 (1.03, 1.09)	.0008	1.05 (1.01, 1.08)	.0166
RFM tertile						
T1	Reference		Reference		Reference	
T2	1.51 (1.03, 2.23)	.0429	1.35 (0.89, 2.03)	.1617	1.26 (0.80, 1.99)	.3244
T3	2.61 (1.68, 4.03)	.0001	2.29 (1.43, 3.69)	.0015	1.96 (1.18, 3.26)	.0169
* P* for trend		.0001		.0018		.0164
WHTR	27.85 (7.56, 102.57)	<.0001	18.48 (4.31, 79.23)	.0004	10.09 (1.95, 52.10)	.0112
WHTR tertile						
T1	Reference		Reference		Reference	
T2	1.51 (1.03, 2.23)	.0429	1.35 (0.89, 2.03)	.1617	1.26 (0.80, 1.99)	.3244
T3	2.61 (1.68, 4.03)	.0001	2.29 (1.43, 3.69)	.0015	1.96 (1.18, 3.26)	.0169
* P* for trend		.0001		.0018		.0164

Model I adjusted for age, race, education level. Model II adjusted for age, race, education level, marital status, PIR, Chol, UA, hypertension, diabetes, ever treated for a pelvic infection/PID, drinking, smoking, age when first menstrual period occurred.

BMI = body mass index, Chol = total cholesterol, C-index = conicity index, CI = confidence interval, PID = pelvic inflammatory disease, PIR = poverty income ratio, RFM = relative fat mass, UA = uric acid, WHTR = waist-to-height ratio.

Subgroup analysis was used to further revealed the relationship between anthropometric indices and infertility (Table 3). None of the stratifications including race, education level, marital status, hypertension, PID, alcohol consumption status, smoke status, PIR, uric acid and age of menarche affected the positive relationship of BMI, C-index, RFM, WHTR and prevalence of infertility. However, we found statistically significant differences with respect to age groups, history of diabetes and level of cholesterol. Our study revealed that age affected the associations between C-index, RFM, WHTR and the prevalence of infertility. C-index tended to be more strongly associated with infertility in all participants (20–45 years), while the relationship between BMI, RFM, WHTR and the prevalence of infertility only found in younger participants (≤30 years). Besides, the history of diabetes affected the positive relationship between RFM with the prevalence of infertility and level of cholesterol affected the positive relationship between BMI with the prevalence of infertility.

**Table 3:** Weighted effect size of BMI, C-index, RFM, WHTR on infertility in prespecified and exploratory subgroups in each subgroup.

Characteristic	BMI		C-index		RFM		WHTR	
	OR (95%CI)	*P*-value	OR (95%CI)	*P*-value	OR (95%CI)	*P*-value	OR (95%CI)	*P*-value
Age (year)								
≤30	1.05 (1.02, 1.08)	.0047	465.58 (21.85, 9921.31)	.0007	1.09 (1.03, 1.14)	.0034	63.19 (6.29, 634.58)	.0019
>30	1.02 (0.99, 1.04)	.1625	13.96 (2.39, 81.47)	.0078	1.03 (0.99, 1.06)	.1120	5.08 (0.90, 28.76)	.0796
* P* for interaction		.0572		.0297		.0274		.0284
Race								
Mexican-American	1.05 (1.00, 1.10)	.0496	38.83 (0.99, 1528.49)	.0658	1.08 (1.00, 1.17)	.0724	45.50 (1.72, 1200.20)	.0339
Other Hispanic	1.03 (0.97, 1.09)	.3910	12.48 (0.03, 4472.57)	.4108	1.05 (0.97, 1.13)	.2475	9.28 (0.12, 700.35)	.3252
Non-Hispanic White	1.03 (1.00, 1.05)	.0555	74.85 (9.94, 563.65)	.0005	1.05 (1.01, 1.09)	.0192	11.83 (1.92, 72.70)	.0153
Non-Hispanic Black	1.01 (0.97, 1.04)	.7112	5.44 (0.25, 118.96)	.2957	1.02 (0.96, 1.08)	.5902	2.47 (0.15, 41.04)	.5347
Other Race	1.02 (0.97, 1.07)	.4046	6.08 (0.03, 1165.89)	.5089	1.03 (0.97, 1.09)	.3355	5.30 (0.14, 199.88)	.3792
* P* for interaction		.7294		.5593		.7347		.6832
Education level								
Less than high school	1.06 (1.01, 1.11)	.0399	127.01 (0.95, 16,956.44)	.0659	1.07 (0.97, 1.18)	.1878	65.88 (1.00, 4327.87)	.0632
High school graduate/GED or equivalent	1.02 (1.00, 1.05)	.1131	4.98 (0.21, 115.68)	.3284	1.03 (0.99, 1.08)	.1196	4.79 (0.71, 32.13)	.1216
More than high school	1.02 (1.00, 1.05)	.1073	54.51 (7.75, 383.25)	.0006	1.05 (1.01, 1.08)	.0255	9.22 (1.60, 52.93)	.0212
* P* for interaction		.3597		.3105		.7552		.4400
Marital status								
Married/living with partner	1.03 (1.01, 1.06)	.0264	75.24 (11.34, 499.31)	.0002	1.05 (1.01, 1.10)	.0174	15.45 (2.32, 102.87)	.0100
Widowed/divorced/separated	1.04 (0.99, 1.09)	.1211	16.74 (0.20, 1425.60)	.2277	1.04 (0.97, 1.12)	.2719	15.59 (0.50, 483.57)	.1319
Never married	0.99 (0.96, 1.03)	.7528	4.29 (0.23, 79.90)	.3401	1.02 (0.98, 1.06)	.3890	1.45 (0.19, 11.01)	.7240
* P* for interaction		.1416		.2275		.3920		.1615
Hypertension								
Yes	1.03 (0.99, 1.06)	.1359	16.52 (0.34, 808.71)	.1718	1.05 (0.98, 1.12)	.1493	10.44 (0.71, 154.09)	.1018
No	1.03 (1.00, 1.05)	.0410	43.46 (6.98, 270.64)	.0005	1.04 (1.01, 1.08)	.0189	10.01 (1.84, 54.37)	.0141
* P* for interaction		.9688		.6450		.8431		.9743
Diabetes								
Yes	1.07 (1.01, 1.13)	.0315	340.80 (0.35, 331,343.77)	.1115	1.16 (1.04, 1.31)	.0164	215.99 (3.30, 14,152.05)	.0199
No	1.03 (1.00, 1.05)	.0343	34.39 (6.20, 190.70)	.0006	1.05 (1.01, 1.08)	.0125	10.37 (1.94, 55.40)	.0124
Borderline	0.91 (0.78, 1.06)	.2391	0.31 (0.00, 238,696.02)	.8657	0.87 (0.70, 1.06)	.1824	0.00 (0.00, 319.94)	.3182
* P* for interaction		.0720		.6184		.0195		.1043
Ever treated for a pelvic infection/PID								
Yes	1.02 (0.97, 1.07)	.5115	177.36 (2.40, 13,107.65)	.0276	1.03 (0.97, 1.11)	.3427	7.70 (0.23, 253.09)	.2641
No	1.03 (1.00, 1.05)	.0373	30.79 (5.38, 176.21)	.0009	1.05 (1.01, 1.08)	.0203	10.42 (1.84, 58.90)	.0145
* P* for interaction		.6869		.4309		.7595		.8712
Alcohol use in the past years								
No	1.04 (1.00, 1.09)	.0651	8.60 (0.13, 551.02)	.3223	1.06 (0.99, 1.14)	.1247	19.62 (0.61, 628.40)	.1072
Yes	1.03 (1.01, 1.05)	.0242	62.17 (12.25, 315.47)	.0001	1.05 (1.02, 1.08)	.0042	12.88 (2.86, 57.87)	.0032
Missing	0.98 (0.90, 1.06)	.6092	2.75 (0.02, 384.20)	.6926	0.97 (0.88, 1.08)	.5925	0.30 (0.00, 147.87)	.7073
* P* for interaction		.3925		.2899		.2661		.4297
Smoke status								
Smoker	1.01 (0.97, 1.05)	.5172	50.07 (3.19, 785.45)	.0111	1.03 (0.97, 1.09)	.3523	5.24 (0.32, 87.01)	.2611
Nonsmoker	1.03 (1.00, 1.06)	.0409	35.32 (4.72, 264.25)	.0023	1.05 (1.01, 1.10)	.0289	13.61 (1.82, 101.79)	.0189
Ex-smoker	1.03 (0.98, 1.07)	.2442	23.07 (0.38, 1410.30)	.1497	1.05 (0.99, 1.12)	.1160	9.46 (0.48, 188.12)	.1555
* P* for interaction		.7304		.9520		.7501		.8115
PIR tertile								
T1	1.01 (0.98, 1.04)	.4522	3.13 (0.27, 35.69)	.3692	1.02 (0.97, 1.06)	.4916	2.33 (0.24, 22.60)	.4741
T2	1.02 (0.99, 1.06)	.2488	164.85 (7.68, 3537.55)	.0039	1.04 (0.99, 1.11)	.1461	11.56 (0.77, 173.32)	.0918
T3	1.04 (1.01, 1.06)	.0184	59.50 (5.27, 672.17)	.0036	1.06 (1.02, 1.10)	.0055	20.77 (3.01, 143.35)	.0059
* P* for interaction		.4877		.0808		.2789		.2971
Chol tertile (mmol/L)								
T1	1.05 (1.02, 1.07)	.0015	32.81 (2.44, 440.99)	.0159	1.08 (1.03, 1.12)	.0016	34.24 (5.42, 216.27)	.0012
T2	1.02 (0.98, 1.05)	.3595	38.75 (2.95, 509.47)	.0115	1.03 (0.98, 1.07)	.3033	4.88 (0.44, 54.44)	.2123
T3	1.01 (0.98, 1.05)	.3729	38.34 (3.84, 382.95)	.0056	1.04 (0.99, 1.08)	.1443	6.03 (0.67, 54.25)	.1245
* P* for interaction		.0466		.9931		.0575		.1084
UA tertile (μmol/L)								
T1	1.05 (1.01, 1.09)	.0131	201.60 (5.07, 8009.89)	.0105	1.08 (1.03, 1.13)	.0045	88.02 (6.55, 1182.99)	.0030
T2	1.02 (0.98, 1.07)	.3596	87.35 (6.09, 1253.76)	.0037	1.02 (0.97, 1.09)	.4262	7.52 (0.39, 146.37)	.1978
T3	1.02 (0.99, 1.04)	.1552	8.32 (0.97, 71.07)	.0670	1.04 (1.00, 1.09)	.0589	5.43 (0.91, 32.40)	.0779
* P* for interaction		.2665		.3005		.2841		.1314
Age when first menstrual period occurred, tertile (yr)								
T1	1.03 (1.00, 1.06)	.1108	16.67 (1.20, 232.03)	.0492	1.05 (1.00, 1.10)	.0889	8.80 (0.90, 86.00)	.0762
T2	1.03 (0.98, 1.07)	.2475	137.54 (3.51, 5388.46)	.0160	1.05 (0.99, 1.12)	.1142	13.35 (0.62, 286.08)	.1131
T3	1.03 (1.00, 1.05)	.0595	28.49 (4.82, 168.51)	.0014	1.04 (1.01, 1.07)	.0161	9.75 (1.95, 48.70)	.0117
* P* for interaction		.9924		.6407		.8795		.9645

Adjusted for age, race, education level, marital status, PIR, Chol, UA, hypertension, diabetes, ever treated for a pelvic infection/PID, drinking, smoking, age when first menstrual period occurred except the subgroup variable.

BMI = body mass index, Chol = total cholesterol, C-index = conicity index, CI = confidence interval, PID = pelvic inflammatory disease, PIR = poverty income ratio, RFM = relative fat mass, UA = uric acid, WHTR = waist-to-height ratio.

### 3.3. C-index showed a good linearly relationship than other markers including BMI, RFM, WHTR for the risk of infertility among all the study participants

**Figure 2.**
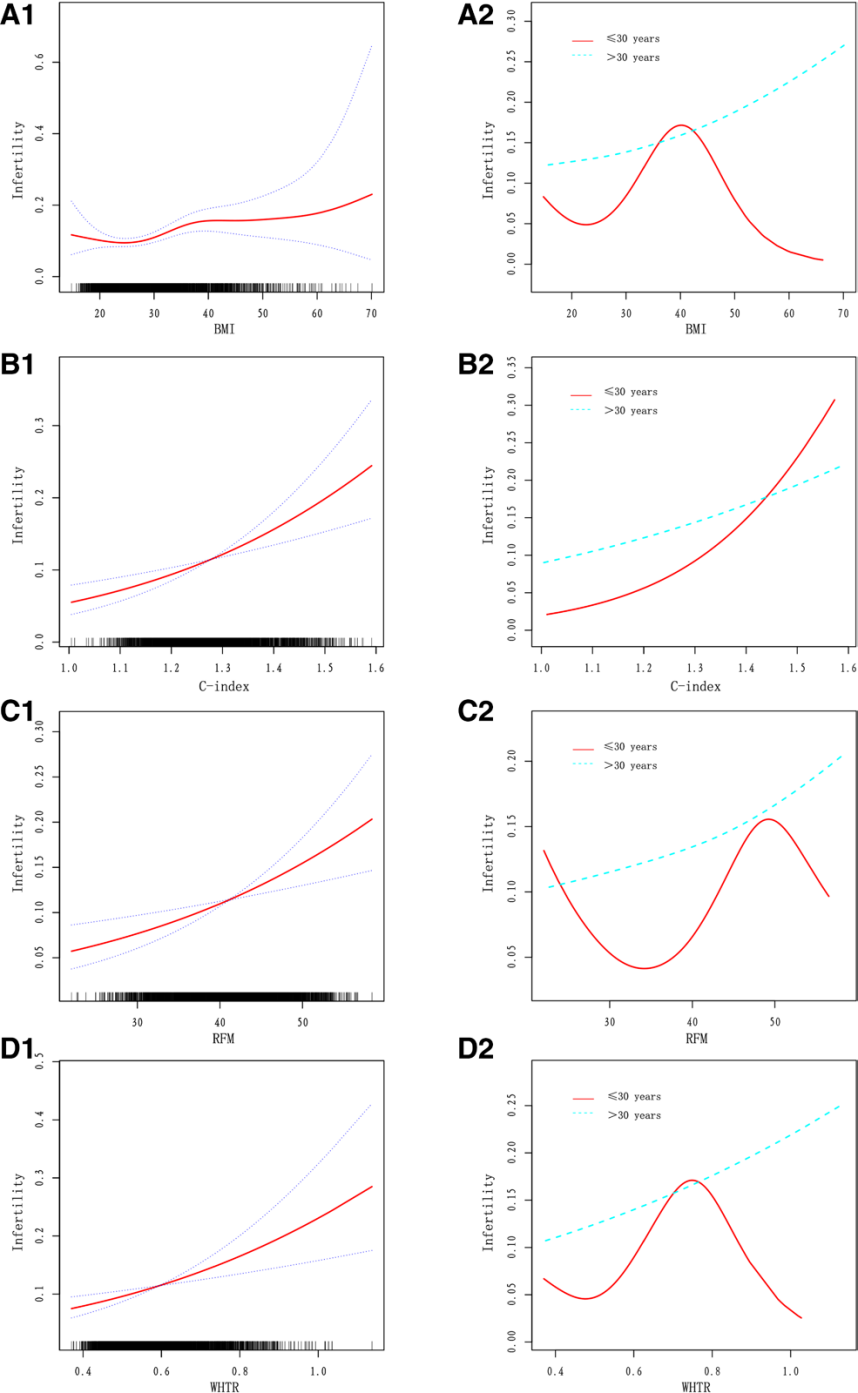
Relationship of anthropometric indices with the risk of infertility for all females and for subgroups of aged. (A1, B1, C1, D1) Dose–response relationship between BMI, C-index, RFM, WHTR and risk of infertility in all females respectively. (A2, B2, C2, D2) Dose–response relationship between BMI, C-index, RFM, WHTR and risk of infertility in females grouped by aged respectively. BMI = body mass index, C-index = conicity index, RFM = relative fat mass, WHTR = waist-to-height ratio.

Smooth curve fitting and stratified analysis were preformed to explore the potential relationship between the anthropometric indices and infertility based on the fully adjusted model. As shown in Figure 2, we found that a good linearly relationship between C-index and infertility among all the study participants, while BMI, RFM and WHTR were found to be positive linearly associated with infertility among study participants over 30 years old. However, nonlinear U-shaped relationship between BMI, RFM, WHTR and infertility were demonstrated among study participants under 30 years old.

### 3.4. C-index demonstrated the better predictive ability than other anthropometric indices

**Figure 3.**
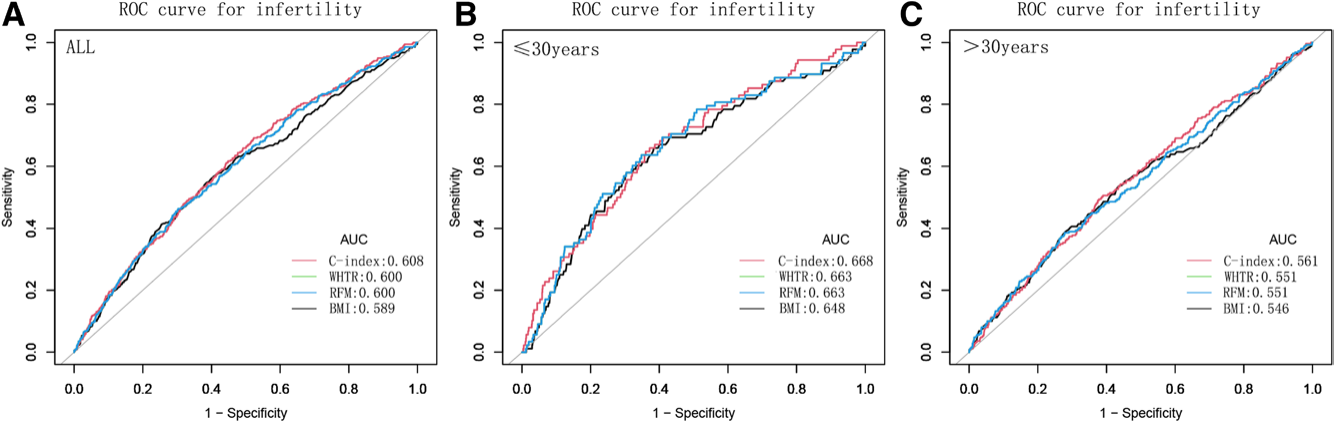
ROC curve analyses to predict infertility. (A) ROC to predict infertility for all female participants. (B and C) ROC to predict infertility for female participants under and over 30 years old. BMI = body mass index, C-index = conicity index, RFM = relative fat mass, ROC = receiver-operating characteristic, WHTR = waist-to-height ratio.

The results of the ROC curve analysis are presented in Figure 3. For predicting the occurrence of infertility, the areas under the curve among all the study participants for BMI, C-index, RFM, and WHTR were 0.589, 0.608, 0.600, and 0.600, respectively (Fig. 3A). Moreover, the AUC values of C-index were remained higher than the other anthropometric indices in predicting infertility after stratified analysis of age, suggesting that C-index maybe have a good advantage in evaluating infertility (Fig. 3B and C).

## 4. Discussion

This study examined the association between anthropometric indices and infertility in non-institutionalized Americans. We found that C-index tended to be more strongly associated with an increased risk of infertility when compared with BMI, RFM, and WHTR in cross-sectional analysis, which included 2948 study participants. The association between C-index and infertility was stable across various demographic situations according to the subgroup analysis and C-index had a good linearly relationship with infertility among all the study participants. In addition, our study showed that C-index was the highest associated with infertility in logistic regression and had the largest AUC in ROC analysis. These research findings indicate that C-index may be a better predictor of infertility than other obesity-related indicators. Our research findings suggest that C-index was a better screening tool for infertility, and managing obesity by C-index may help to reduce the risk of infertility.

Previous studies have demonstrated a close relationship between obesity and infertility. According to a prospective cohort study of black women, a higher waist circumference (WC > 84 cm) or Waist-to-Hip Ratio (WHR > 0.85), that relate with abdominal fat distribution, was associate with lower fecundity independently of BMI.^[40]^ Several studies have demonstrated that the patterns of fat distribution and body composition parameters are related to ovarian failure including primary amenorrhea, polycystic ovary syndrome (PCOS), and anorexia nervosa.^[41]^ Besides, Wang et al discovered that fat distribution varies among infertile women, and higher amounts of fat are associated with poorer assisted reproductive outcomes by performing a prospective cohort study included 576 infertile women.^[42]^ However, it is difficult to determine body fat composition accurately for it needs the professional equipment and trained health technicians. C-index is a superior anthropometric indicator compared to BMI for accessing the distribution of body fat, especially abdominal adiposity.^[43]^ C-index has been proved a good predictor for future diabetes, cardiovascular disease and mortality.^[44,45]^ However, research focusing on the C-index and its relationship with infertility is limited.

Recently, increasing attention has been given to the relationship between anthropometric index and infertility. Previous studies have pointed out that the association between infertility and BMI followed a U-shaped pattern, indicating that both extremely low and high BMI values were linked to an increased risk of infertility. RFM has been developed as a new indicator of whole-body fat percentage. According to a cross-section study of Correa et al, RFM is more accurate to diagnose obesity when compared to BMI.^[46]^ Ashwell et al showed that WHTR can be more predictive than BMI as early indicator of obesity risks.^[47,48]^ However, a multiethnic prospective cohort found no correlations between WHTR with fecundability.^[49]^ In this study, we found significant associations for BMI, C-index, RFM and WHTR with infertility and the associations between C-index and infertility were most pronounced.

This study is based on the NHANES data of female aged 20 to 45 years to investigate the correlations between anthropometric indices and infertility risk in the non-institutionalized Americans. Consistent with several previous studies, our findings also demonstrated the association between BMI, WHTR and infertility. Besides, we first showed the association between C-index, RFM and infertility. Our research findings suggest that C-index outperforms BMI, RFM and WHTR in predicting infertility. To our knowledge, this is the first research to explore the association between C-index and infertility, highlighting the association between a higher C-index level and increased risk of infertility. Given that obesity has a negatively impact on infertility, utilizing C-index as a simple yet accurate tool for evaluating the risk of infertility may be a better option.

However, there are several limitations that need to be declared. First, the date of our study included 2948 females living in the US, aged 20 to 45 years. Therefore, the findings may not apply to females outside this age or living outside the US. Second, we were unable to draw a causal relationship between anthropometric indices and female infertility due to the cross-sectional design of the NHANES. Third, since the lack of information on the specific causes of infertility in the NHANES database, some of the infertility females included in this study may resulted from male factors. Additionally, despite analyzing several infertility-related factors, some were still not considered in our study. Thus, the conclusions of this study should be interpreted with caution.

In conclusion, the findings of this study indicated that elevated levels of C-index were strongly linked to the risk of developing infertility U.S. adults, suggesting that C-index potential use in predicting and managing infertility. However, more well-designed studies are needed to further confirm the causal relationship between C-index and infertility.

## Acknowledgments

We thank the assistance of all authors in the manuscript.

## Author contributions

**Conceptualization:** Shao-Cong Liang.

**Formal analysis:** Jia-Yi Zhao, Meng-Hui Hong.

**Investigation:** Xiao-Ming Yang, Tao Zhou.

**Resources:** Meng-Hui Hong.

**Supervision:** Shao-Cong Liang.

**Writing – original draft:** Jia-Yi Zhao.

**Writing – review & editing:** Jia-Yi Zhao.
